# Kibble diet is associated with higher faecal glucocorticoid metabolite concentrations in zoo-managed red wolves (*Canis rufus*)

**DOI:** 10.1093/conphys/coae008

**Published:** 2024-02-27

**Authors:** Morgan Bragg, Carly R Muletz-Wolz, Nucharin Songsasen, Elizabeth W Freeman

**Affiliations:** Environmental Science and Policy Department, George Mason University, 4400 University Dr. Fairfax, VA 22030, USA; Center for Conservation Genetics, Smithsonian National Zoo & Conservation Biology Institute, 3001 Connecticut Ave. NW Washington, DC, 20008 USA; Center for Species Survival, Smithsonian National Zoo & Conservation Biology Institute, 1500 Remount Road, Front Royal, VA 22630, USA; Center for Conservation Genetics, Smithsonian National Zoo & Conservation Biology Institute, 3001 Connecticut Ave. NW Washington, DC, 20008 USA; Center for Species Survival, Smithsonian National Zoo & Conservation Biology Institute, 1500 Remount Road, Front Royal, VA 22630, USA; School of Integrative Studies, George Mason University, 4400 University Dr. Fairfax, VA 22030, USA

**Keywords:** Red wolf, fecal glucocorticoid metabolites, diet, zoo management, animal welfare

## Abstract

The red wolf (*Canis rufus*) is a critically endangered canid that exists solely because of the establishment of the *ex situ* population in the late 1980s. Yet, the population under human care suffers from gastrointestinal (GI) disease in captivity. While the cause of GI disease is unknown, it is speculated that environmental factors can influence GI health of zoo-managed red wolves. The goal of the present study was to investigate the relationship between faecal glucocorticoid metabolite (FGM) concentrations, a biomarker for stress, and environmental factors for zoo-managed red wolves. Faecal samples were collected from 14 adult wolves three times a week for 5 to 12 months. Using a single-antibody cortisol enzyme immunoassay, FGM concentrations were quantified. Environmental factors were collected for each participating wolf on dietary type, sex, type of public access to enclosure, density (enclosure size [ft^2^]/number of wolves living in enclosure) and a monthly average status of GI health. Red wolves that ate a commercial kibble diet had both higher FGM concentrations over time and higher baseline FGM concentrations compared to individuals that received commercial kibble mixed with commercial meat. Density, public access or GI health were not related to FGM concentration; however, males had higher baseline FGM concentrations compared to female red wolves. Our findings suggest that management conditions, particularly diet, can strongly influence FGM concentration in the zoo-managed red wolf population. Findings from this study highlight the importance of management choices on individual welfare. Maintaining a healthy captive population of red wolves is imperative for the persistence of the species, including successful future reintroductions.

## Teaser

Suboptimal health is a main challenge in managing wildlife populations under human care. We found that kibble-only diets are linked to higher faecal glucocorticoid metabolite concentrations in zoo-managed red wolves. A deficient diet can likely act as a stressor and risk factor for poor health.

## Introduction

The American red wolf (*Canis rufus*) is a critically endangered canid that once was nearly extirpated from its historic range in the southeastern United States due to persecution by humans (Hinton *et al.,* 2013). Today, there are ~250 red wolves left in existence; 90% of those individuals live under human care (U.S. Fish and Wildlife Service, 2022), which emphasizes the importance of *ex situ* populations. Recently, United States Fish and Wildlife Services (USFWS) resumed reintroduction of red wolves into Alligator River National Wildlife Refuge in Manteo, North Carolina. From 2021 to 2023, USFWS released 16 adults and plan for more in the future (U.S. Fish and Wildlife Service, 2023). Therefore, maintaining sustainable and healthy *ex situ* populations is the key to the success of the reintroduction programme.

In mammals, the *ex situ* environment can trigger an adrenal response and increased glucocorticoid production due to enclosure type and size, diet and proximity to conspecifics or heterospecifics, including humans (Keay *et al.,* 2006; Cavigelli and Chaudhry, 2012; van Kesteren *et al.,* 2012; McKenzie *et al.,* 2017). Although short-term elevation of glucocorticoids is necessary for an individual to survive a stressor (Keay *et al.,* 2006), prolonged activation of the hypothalamic pituitary adrenal (HPA) axis (chronic stress) can compromise health and reproduction (de Weerth, 2017; Lattin and Kelly, 2020). No standard hormone profile of a chronically stressed individual exists. However, consistently elevated glucocorticoid concentrations are generally regarded as being indicative of a stressed animal (Palme, 2019; Romero and Gormally, 2019). Previous studies in wild animals reported impacts to reproduction and health (Creel, 2005; Young *et al.,* 2006; Sheriff *et al.,* 2011; Lattin and Kelly, 2020) in individuals with consistent heightened glucocorticoid, which is why we are interested in how management conditions impact faecal glucocorticoid metabolite (FGM) concentrations, a biomarker for stress, in the zoo-housed red wolves.

Diet also can influence glucocorticoid production and metabolism. Stetz *et al.* (2013) found that wild bears that have eaten a diet with higher nutritional value have lower FGM concentrations than bears eating vegetation with lower nutritional value, indicating that diet composition and quality can impact glucocorticoid production. A high-fibre diet could make transit time faster in the gut that, in turn, could decrease the amount of time for glucocorticoids to be reabsorbed by the intestines, leading to increased FGM excretion (von der Ohe *et al.,* 2004; Keay *et al.,* 2006). Furthermore, differences in diet composition can produce different glucocorticoid metabolites and the antibody cross-reactivity of each metabolite can vary, possibly influencing detectability (Goymann, 2012). In the wild, the red wolf diet consists of white tail deer (*Odocoileus virginianus*), rodents (*Rodentia* spp.), rabbits (*Lagomorpha* spp.), raccoon (*Procyon lotor*) and other small mammals (McVey *et al.,* 2013). In zoos, the daily diet consists of 90–95% meat-based dry kibble for domestic dog and 5–10% supplemental items (e.g. bones, commercial meat and carcass) (Association of Zoos and Aquariums Canid Taxonomic Advisory Group, 2012). The kibble diet contains a large amount (30–60%) of starch (Fortes *et al.,* 2010) that wolves are not adapted to digesting (Axelsson *et al.,* 2013). In addition, there are some facilities that feed a mixed-diet daily—a mix of kibble and commercial meat. As it has been shown that diet can influence glucocorticoid production, we hypothesized that the differences in diet offered among institutions would result in variations in HPA axis activation and FGM concentrations in zoo-managed red wolves.

The amount of available space for individuals within an enclosure, or density, and public access to enclosures also can influence HPA axis activation. Several studies reported a relationship between FGM concentrations and enclosure size and height in felids (Wielebnowski *et al.,* 2002; Quirke *et al.,* 2012; Vaz *et al.,* 2017). Furthermore, FGM concentrations have been observed to increase as the number of zoo visitors increased in the blackbuck (*Antilope cervicapra*), spider monkey (*Ateles geoffroyii rufiventris*) and clouded leopard (*Neofelis nebulosa*) (Wielebnowski *et al.,* 2002; Davis *et al.,* 2005; Rajagopal *et al.,* 2011). In contrast, a previous study of 13 red wolves did not find differences in FGM concentrations between wolves on and off exhibit (Franklin *et al.,* 2020). However, based on the wide-ranging and elusive nature of the species, we hypothesized that higher density and higher frequency of visitors would be linked to higher FGM in the red wolf.

Even though management of red wolves in zoos has been critical to their long-term survival, it is tied to health disorders not described in wild populations (Acton *et al.,* 2000; Association of Zoos and Aquariums Canid Taxonomic Advisory Group, 2012). Unfortunately, gastrointestinal (GI) disease caused death in 21% of adult captive red wolves from 1992 through 2012 (Acton *et al.,* 2000; Seeley *et al.,* 2016). Common signs of GI disease in red wolves include chronic diarrhoea and intestinal inflammation, making it difficult to absorb nutrients (Association of Zoos and Aquariums Canid Taxonomic Advisory Group, 2012). The cause of GI disease is unknown, but there is evidence that alteration in the composition of the gut bacterial community can lead to GI health issues, which could be caused by factors like diet, infection, antibiotics or genetics (Petersen and Round, 2014; Jandhyala *et al.,* 2015; Bragg *et al.,* 2020). Furthermore, shifts in gut bacterial community composition and intestinal barrier permeability can be influenced by glucocorticoid production (Davidson *et al.,* 2018). Understanding the relationship between environmental factors and FGM concentrations could illuminate possible contributors to GI disease, which could be observed non-invasively through faecal consistency score, in the *ex situ* red wolf population. Due to the specialized equipment and expertise needed to diagnosis GI disease, faecal consistency score has been used as a proxy for GI health status in the domestic dog (Niina *et al.,* 2019; Bermingham *et al.,* 2017; Jergens *et al.,* 2003) and human (Falony *et al.*, 2019) but has not been validated in the red wolf, thus making it a potential parameter for estimating GI disease status for this species.

The objective of this study is to understand if there is a link between FGM concentrations and environmental factors in the zoo-managed red wolves. We hypothesized that poor GI health status, a kibble diet, higher density and self-guided public access will be associated with increased glucocorticoid production in zoo-managed red wolves. Overactivation of the HPA axis from environmental stressors can support the evolution of GI pathologies (O’Mahony *et al.,* 2009; [Bibr ref11][Bibr ref11]; de Weerth, 2017; Sylvia and Demas, 2018; Cryan *et al.,* 2019). Findings from this study will help decipher the relationship between FGM concentrations and environmental factors, aiding in the identification of stressors that could be acting as risk factors for GI disease in red wolves.

## Materials and Methods

### Animals and sample collection

We collected data from 14 red wolves across seven zoological institutions in the USA (Table 1). Faecal samples were opportunistically collected three times a week for 5 to 12 months. Samples were collected by facility staff and kept at −20°C until shipment to Smithsonian National Zoo & Conservation Biology Institute. For wolves housed in groups, identification of samples from individual wolves was done by feeding markers such as plastic beads or food dye (depending on each institution’s preference).

**Table 1:** Fourteen participating red wolves, including their facility, sex, diet type, type of public access, density (enclosure size [ft^2^]/number of wolves living in enclosure) and average canine inflammatory bowel disease activity index (CIBDAI) score of each wolf

**Animal**	**Facility**	**Sex**	**Diet type**	**Public access**	**Density**	**Average CIBDAI (±SEM)**
RW1	A	F	Mixed	Staff	20 984	0 **±** 0
RW2	B	F	Kibble	Staff	5500	1.1 **±** 0.13
RW3	B	M	Kibble	Staff	5500	1.1 **±** 0.12
RW4	C	F	Mixed	Self	3550	0 **±** 0
RW5	D	M	Mixed	Self	4500	0.5 **±** 0.14
RW6	E	M	Mixed	Self	8100	2 **±** 0
RW7	F	F	Mixed	None	2500	1.2 **±** 0.49
RW8	F	F	Kibble	None	2500	0 **±** 0
RW9	F	F	Kibble	None	2500	0 **±** 0
RW10	F	F	Kibble	None	2500	0.3 **±** 0.16
RW11	G	F	Mixed	Staff	8712	0 **±** 0
RW12	G	F	Mixed	Staff	10 890	0 **±** 0
RW13	G	M	Mixed	Staff	8712	0 **±** 0
RW14	G	M	Mixed	Staff	10 890	0 **±** 0

Environmental information including dietary type (kibble or mixed), relation of housing group (single, breeding pair or family group), type of public access to enclosure (none, self or staff guided), density (enclosure size [ft^2^]/number of wolves living in enclosure) and monthly canine inflammatory bowel disease activity index (CIBDAI) was gathered for each participating facility (Supplementary Material). Individuals were fed diets composed of a meat-based dry kibble approved for domestic dogs (kibble) or a mix of kibble and commercial meat (mixed). Density was measured, rather than enclosure or group size, because it is a better measure of the amount of space that is available for each wolf to use. The CIBDAI is a standardized assessment for clinical signs of GI disease in domestic dogs (Jergens *et al.,* 2003), and scores attitude/activity, appetite, vomiting, stool consistency, stool frequency and weight loss on a scale of zero to three, with three being the most severe. The ratings of the six signs are added up to give a total CIBDAI score; 0–3 implies clinically miniscule disease, 4–5 implies mild presence of disease, 6–8 indicates moderate presence of disease, and 9 or higher indicates severe presence of disease.

### Glucocorticoid extraction

Faecal samples were lyophilized, crushed and sifted prior to FGM extraction. Steroid extraction was performed using a modified method of that published by Young *et al.* (2004). Briefly, 0.2 g (±0.02 g) of lyophilized faecal powder was shaken for 30 min in 90% ethanol. Samples were centrifuged at 1500 rpm for 20 min and the first supernatant was recovered. The remaining pellet was resuspended in 5 mL of 90% ethanol, centrifuged again at 1500 rpm for 15 min and the second supernatant was recovered, combined with the first supernatant and dried down under air. Once dried down, it was resuspended in 100% methanol and allowed to air dry and then suspended in phosphate buffer saline (0.2 M NaH_2_PO_4_, #S8282; 0.2 M Na_2_HPO_4_, #S7907, Sigma Aldrich; 0.15 M NaCl, #S271, Fisherbrand; pH 7.0) and stored at −20°C until utilized for hormone assays. Steroid extraction efficiencies were determined with the addition of radiolabeled hormone (^3^H-cortisol; 4000–8000 dpm) and average recovery after extraction was 81% for all samples.

### Enzyme immunoassay

We used an in-house cortisol enzyme immunoassay (EIA; R4866, Munro, University of California, Davis, CA; 1:85 [C.J. Munro, University of California, Davis]) as previously described by our laboratory (Young *et al.,* 2004; Maly *et al.,* 2018; Putman *et al.,* 2019; Fazio *et al.,* 2020; Maly *et al.,* 2021; Yu *et al.,* 2021). All samples were run in duplicates at the same time. Inter-assay variation was <15% and intra-assay variation was <10%. The coefficient of variation was calculated by dividing standard deviation of the percent binding by the mean of the percent binding then multiplying by 100 (Brown *et al.,* 2008). Assay sensitivity was 0.039 ng/g faeces. Serial dilutions of faecal extracts yielded a displacement curve parallel to the standard curve (*y* = 1.261*x* − 15.559, *R*^2^ = 0.975, *F*_1,5_ = 161.905, *P* < 0.001).

### Statistical analyses

We conducted all statistical analyses in R (version 4.1.2) (R Core Team, 2021). Using the function ‘hormBaseline’ in the package ‘hormLong’ (Fanson and Fanson, 2015), baseline and peak FGM concentrations (+1.5SD above mean) (Wielebnowski *et al.,* 2002; Edwards *et al.,* 2015; Franklin *et al.,* 2020) of FGM were identified for each individual. Baseline is defined as the concentration of glucocorticoids required for normal physiological function (Bonier *et al.,* 2009). Average FGM concentrations were calculated each week for each wolf to account for uneven sampling frequency among individuals (Jones *et al.,* 2018).

Linear mixed models were used to assess relationships between FGM concentrations and environmental factors utilizing the function ‘lmer’ in the ‘lme4’ package (Bates *et al.,* 2015). We included weekly averages of FGM concentrations as the response variable and sex, public access, diet type, density (enclosure size [ft^2^]/number of wolves living in enclosure) and monthly CIBDAI score as explanatory variables. Sex was included to evaluate if it was a factor driving variation seen in FGM concentration. We used backwards model selection using the ‘step’ function in the *lmerTest* package (Kuznetsova *et al.,* 2017). First, we determined the appropriate random effect of facility, animal or animal nested within facility with restricted maximum likelihood (REML) on the full model and compared the models with AIC using the ‘step’ function in the ‘lmerTest’ package. Those results indicated that animal and facility were the appropriate random effects to account for repeated sampling from the same individuals at different zoos. Then, a full model with the appropriate random effects and all fixed effects of interest (sex, public access, diet type, density and monthly CIBDAI) was run with REML set to false to determine the significance of the explanatory variables. Linear mixed models fit by REML log-likelihood *t*-test used Satterthwaite approximations to degrees of freedom. We used ‘VIF’ function in the ‘regclass’ package (Petrie, 2020) to assess correlations between explanatory variables. Variables with a VIF value of greater than 5 are considered correlated and excluded from being in the same model. None of the explanatory variables were correlated in this study.

We ran an additional linear mixed effect model to assess the relationship between the baseline FGM concentration of each individual and environmental factors using the ‘lmer’ function in the ‘lmerTest’ package. We included baseline FGM concentrations as the response variable and sex, public access, diet type, density and monthly CIBDAI score as explanatory variables with REML set to false to determine the significance of the explanatory variables. We included facility as a random effect to account for variation across the different institutions. Similarly, we used backwards model selection using the ‘step’ function in the ‘lmerTest’ package. Statistical significance was set at alpha equals 0.05.

## Results

A total of 899 faecal samples were collected from 14 wolves (9 females, 5 males) across 7 facilities. The median FGM concentration was 258 ng/g and ranged from 2 to 8089 ng/g (Table 2). The average baseline sample ranged from 71 to 791 ng/g and average peak samples (+1.5SD) ranged from 223 to 2031 ng/g (Table 2).

**Table 2:** Faecal glucocorticoid metabolite (FGM) results from 14 red wolves managed in zoos, including the sample size (*n*), mean plus/minus standard error (SEM), median, minimum and maximum raw FGM concentrations (ng/g), as well as baseline samples and mean of the peak samples (+1.5SD; ng/g)

**Animal**	***n***	**Mean ± SEM**	**Median**	**Min**	**Max**	**Baseline**	**Peak mean**
RW1	26	824 ± 131	640	176	2793	238	1085
RW2	143	202 ± 12	168	49	1127	71	223
RW3	132	230 ± 11	213	37	751	119	291
RW4	51	259 ± 37	206	38	1797	173	539
RW5	39	321 ± 65	187	35	2198	137	586
RW6	76	219 ± 29	165	29	1887	103	356
RW7	30	155 ± 18	129	35	447	117	309
RW8	82	1179 ± 85	1029	271	5877	791	1785
RW9	89	1475 ± 153	1003	34	8090	380	2031
RW10	62	1034 ± 79	952	157	3703	624	1532
RW11	49	203 ± 14	186	9	481	152	321
RW12	38	506 ± 86	353	30	2717	237	1085
RW13	39	468 ± 51	369	74	1322	230	720
RW14	43	318 ± 42	247	2	1665	198	593

**Figure 1:**
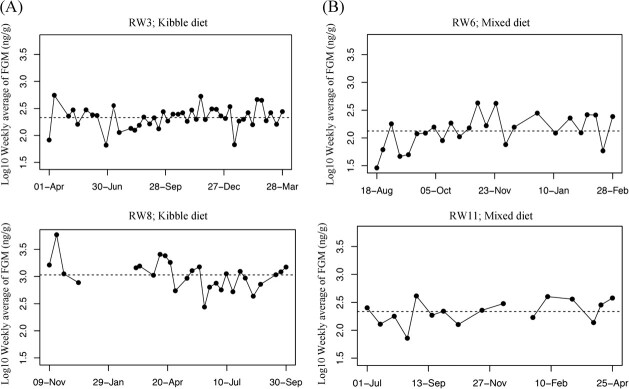
Longitudinal profiles of log10 weekly average faecal glucocorticoid metabolite (FGM) concentrations comparing a red wolf that ate kibble (**A**) versus mixed diet (**B**). Dashed line represents the mean log10 weekly average faecal glucocorticoid metabolite concentrations for each individual.

The best model on the relationship between FGM and sex, public access, diet type, density and monthly CIBDAI score indicated that diet type was the sole explanatory factor linked to FGM concentrations (*t*_20.33_ = −6.34, *P* value < 0.0001; Table 3; Supplementary Table 1). We found that wolves that ate a mixed diet (mean ± SE = 331 ± 29.10 ng/g) had lower FGM concentrations than wolves that ate a kibble diet (mean ± SE = 691 ± 68.64 ng/g) (Fig. 1; Supplementary Fig. Fig. 1). The fixed effect in the model accounted for 38% of the variation while the fixed and random effects in the model accounted for 72% of the variation in weekly average FGM concentrations (Table 3). Density was not included in the best model; however, there was some indication of a positive relationship between the variable and weekly average FGM concentrations (Supplementary Table 1). No relationship was detected between weekly averages of FGM concentrations and the monthly CIBDAI score, sex or public access, respectively (Supplementary Table 1).

**Table 3:** Estimates of coefficients of the best linear model investigating the impact of environmental variables on the log10-transformed weekly average FGM concentrations of red wolves (*n* = 14)

Log10 weekly average FGM concentrations (ng/g)			
*Predictors*	*Estimates*	*CI*	*P*
(Intercept)	2.96	2.66–3.26	**<0.001**
Diet type [mixed]	−0.71	−0.93 to −0.49	**<0.001**
**Random effects**			
*σ* ^2^	0.09		
^τ^00 Animal	0.00		
^τ^00 Facility	0.11		
ICC	0.55		
*N* Animal	14		
*N* Facility	7		
Observations	301		
Marginal *R*^2^/conditional *R*^2^	0.378/0.723		

Bold indicates *P* < 0.05. For diet type, kibble (intercept) is compared to mixed

**Figure 2:**
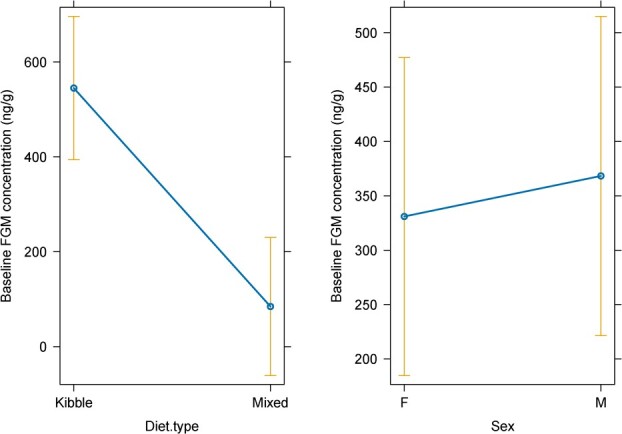
Effects of diet type (left) and sex (right) on baseline faecal glucocorticoid metabolite (FGM) concentrations in red wolves (*n* = 14). Means (± standard error) in each treatment combination calculated from generalized linear mixed-effects models, controlling for random effects.

Diet type and sex had the strongest influence on baseline FGM concentrations in zoo-managed red wolves (Diet type: *t*_300.04_ = −15.1, *P* value < 0.001; Sex: *t*_297.55_ = 2.48, *P* value = 0.01; Table 4). We found that female wolves and wolves that ate a mixed diet had lower baseline FGM concentrations compared to male red wolves and wolves that ate a kibble diet (Fig. 2). The fixed effects accounted for 52% of the variation while the fixed and random effects in the model accounted for 91% of the variation in baseline FGM concentrations (Table 4). No relationship was detected between baseline FGM concentrations and public access, density and monthly CIBDAI score.

**Table 4:** Estimates of coefficients of the best linear model investigating the impact of environmental variables on baseline FGM concentrations of red wolves (*n* = 14)

Baseline FGM concentrations (ng/g)			
*Predictors*	*Estimates*	*CI*	*P*
(Intercept)	531.41	379.73–683.09	**<0.001**
Diet type [mixed]	−460.56	−520.60 to −400.52	**<0.001**
Sex [M]	37.31	7.64–66.98	**0.014**
**Random effects**			
*σ* ^2^		8518.50	
^τ^00 Facility		37 450.15	
ICC		0.81	
*N* Facility		7	
Observations		301	
Marginal *R*^2^/Conditional *R*^2^		0.524/0.912	

Bold indicates *P* < 0.05. For diet type, kibble (intercept) is compared to mixed and for sex, female (intercept) is compared to male

## Discussion

It is known that environmental stressors can initiate activation of the HPA axis and influence glucocorticoid production that is required to maintain homeostasis. However, inappropriate or overactivation of the HPA axis can negatively influence the GI health of an individual (O’Mahony *et al.,* 2009; Brzozowski *et al.,* 2016; de Weerth, 2017; Sylvia and Demas, 2018; Cryan *et al.,* 2019). Thus, we wanted to investigate the link between environmental factors and HPA axis activation, detected via FGMs, that could negatively impact GI health in the red wolf. In the present study, we reported that dietary type (kibble or mixed) was the most influential predictor variable of FGM excretion. Specifically, red wolves that ate a daily diet of kibble had higher FGM concentrations than individuals that ate a daily diet of commercial meat mixed with kibble. Relationships between FGM concentrations and sex, public access and monthly CIBDAI score were not apparent. We did observe a potential effect of density on weekly average FGM concentrations. Moreover, we documented higher baseline FGM concentrations in males and in wolves that ate a daily diet of kibble. No relationships were detected between baseline FGM concentration and public access, density and monthly CIBDAI score. Our objective was to conduct a broad observational study testing for the relationship of multiple factors with FGM concentrations, and we suggest that these factors, particularly diet and density, warrant future studies with greater replication.

We documented a relationship between kibble diet and high FGM concentrations. The main diet of free ranging red wolves consists of white tail deer, small mammals and rodents (Dellinger *et al.,* 2011). However, meat-based kibble formulated for domestic dog are commonly offered to zoo-managed red wolves (Association of Zoos and Aquariums Canid Taxonomic Advisory Group, 2012). Although the kibble is meat based, it also is 30–60% carbohydrates, which provides energy and fibre (Fortes *et al.,* 2010). Unlike domestic dogs, wolves cannot digest starch, a type of carbohydrate, due to differences in the gene responsible for producing the enzyme required to break down starch (Axelsson *et al.,* 2013). Dogs have had thousands of years to evolve the ability to digest a high-carbohydrate diet, but red wolves have only been in zoos for ~50 years (U.S. Fish and Wildlife Service, 2022), reducing their ability to effectively use carbohydrates. Recently, we reported zoo-managed red wolves that ate a kibble diet had different gut microbiome composition, or collection of bacteria in the gut, and increased abundance of two bacterial taxa associated with carbohydrate fermentation compared to wolves that ate a mixed, whole meat (carcass) or wild diet (Bragg *et al.,* 2020). The kibble diet presented in this study contains 4–5% fibre while the mixed diet is a 50/50 combination of kibble and commercial meat that contains only 1% fibre, thus reducing the total amount of carbohydrates and fibre present. The impact of diet composition and quality on FGM concentrations has been established in other species like the black footed ferret (*Mustela nigripes*) (Santymire *et al.,* 2020) and wild gull-billed tern chicks (*Gelochelidon nilotica*) (Albano *et al.,* 2015).

Although the cause of increased FGM concentrations in red wolves fed kibble have not been elucidated, it could be that the rise in hormone concentration may partly be due to an increased defecation frequency that results from the higher amount of fibre in the diet (Palme, 2019), a difference in the type and detection of glucocorticoid metabolites between the two dietary types (Goymann, 2012) or it truly could be an increase in activation of the HPA axis. Higher FGM concentrations are associated with a diet high in fibre in wild Alaskan brown bears (*Ursus arctos horribilis*; von der Ohe *et al.,* 2004), North American red squirrels (*Tamiasciurus hudsonicus*; Dantzer *et al.,* 2011), zoo-managed spider (*Ateles spp.*) and woolly monkeys (*Lagothrix* ssp.; Ange-van Heugten *et al.,* 2009). A diet high in fibre could decrease gut passage time, which decreases time for reabsorption of glucocorticoids by the intestines, thus increasing FGM concentrations (von der Ohe *et al.,* 2004; Keay *et al.,* 2006; Dantzer *et al.,* 2011). It is possible that alterations in gut transit time can influence distribution of nutrients to gut bacteria (Carabotti *et al.,* 2015), a risk factor for poor GI health. It is also possible that differences in diet content can influence gut microbiome composition, leading to the production of various glucocorticoid metabolites via different bacterial taxa present in the gut of wolves eating a kibble compared to a mixed dietary type (Goymann, 2012).

We documented a relationship between high baseline FGM concentrations and male red wolves. Our results agree with Escobar-Ibarra *et al.* (2017) who also reported higher FGM concentrations in male Mexican grey wolves (*Canis lupus baileyi*) compared to females. Red wolves in the wild form multigenerational packs where all members contribute to the maintenance and upbringing of the pack. Pack dynamics or social stress can invoke variations in glucocorticoid production (Cavigelli and Chaudhry, 2012). For example, higher FGM concentrations were observed in dominant individuals compared to subordinate individuals in large canids, suggesting that social status can impact glucocorticoid production (Creel, 2005; van Kesteren *et al.,* 2012; Escobar-Ibarra *et al.,* 2017). Also, social stress can influence factors like reproduction in canids, which is a top priority for a critically endangered species (Kleiman, 2011; Yordy and Mossotti, 2016). It is possible that the higher baseline FGM concentrations we documented in males are a result of social dynamics and/or biological differences between the sexes.

In the present study, we found no relationship between CIBDAI and FGM concentrations. The average monthly CIBDAI score of wolves in this study was 0.44, representing clinically miniscule disease, making it difficult to explore the relationship between CIBDAI and FGM concentrations. The CIBDAI was developed using histology and laboratory observations of intestinal inflammation and is used in domestic dogs as a scoring system to assess clinical activity of canine GI disease (Jergens *et al.,* 2003). It is possible that CIBDAI may not be suitable as a proxy for GI health in red wolves as we know that red wolves in captivity are prone to GI disease via histopathology (Seeley *et al.,* 2016; Acton *et al.,* 2000). Thus, future studies will require greater sampling of animals known to have poor GI health and combining histopathology and various biomarkers for poor GI health in canids like faecal alpha-1 proteinase inhibitor, serum folate and cobalamin (Murphy *et al.,* 2003; Heilmann and Steiner, 2018) to determine the relationship between chronic stress and GI health in zoo-managed red wolves.

Considering the importance of maintaining a sustainable and healthy *ex situ* population, future studies involving a larger sample size with equal distribution among explanatory variables are required to confirm the relationship between diet and glucocorticoids in the red wolf. Furthermore, we recognize that monitoring FGM concentrations is only one piece of information that is involved in the complex stress response. Therefore, additional studies should conduct longitudinal monitoring of multiple components concurrently, like FGM concentrations, behaviour ([Bibr ref39][Bibr ref39]) and secretory immunoglobulin A (Sheriff *et al.,* 2011), to fully assess the response to an environmental stressor. Moreover, it is possible that shifts in the gut microbiome that are related to poor GI health may also alter metabolism of hormones, further impacting FGM concentrations (De Palma *et al.,* 2014). If the shift in gut microbiome composition due to poor GI health could alter metabolism of FGMs, then it would be expected that FGM concentrations would be correlated with biomarkers of GI health, potentially even CIBDAI.

In conclusion, the findings from the present study documented higher FGM concentrations in zoo-managed red wolves that ate a kibble diet compared to mixed diet. In addition, we documented higher baseline FGM concentrations in males and in wolves that ate a kibble diet. Density also showed a potential link to FGM concentrations that warrants future research. These data generated in the current study can be used to better understand general baseline and peak FGM concentrations and the impacts that husbandry practices have on HPA axis activation to provide targeted management recommendations for zoo-housed red wolves. We acknowledge the effort and limitations present in zoo facilities. Thus, it is suggested to reduce the amount of kibble and increase the amount of meat (commercial or whole carcass) in the daily diet of zoo-managed red wolves if possible. Chronic stress can predispose individuals to health issues, but additional studies are needed to confirm this relationship in the red wolf. A greater understanding of the relationship between chronic stress, environmental factors and GI health will help enhance the management of this critically endangered species to ensure its long-term survival.

## Supplementary Material

Web_Material_coae008

## Data Availability

The data underlying this article will be shared on reasonable request to the corresponding author.
