# Supplementary material for: Kibble diet is associated with higher faecal glucocorticoid metabolite concentrations in zoo-managed red wolves (Canis rufus)
Source: Conserv Physiol. 2024 Feb 27;12(1):coae008. doi: 10.1093/conphys/coae008 (PMC10898788; doi:10.1093/conphys/coae008)
Supplement: Web_Material_coae008 [file web_material_coae008.zip › Supplementary material.pdf]

Supplementary material

Kibble diet is associated with higher fecal glucocorticoid metabolite concentrations in zoo-managed red wolves (*Canis rufus*)

Morgan Bragg[1,2,3], Carly Muletz-Wolz[2], Nucharin Songsasen[3], Elizabeth W. Freeman[4]

[1]Environmental Science and Policy Department, George Mason University, Fairfax, 22030, USA

[2]Center for Conservation Genetics, Smithsonian National Zoo & Conservation Biology Institute, Washington, D.C. 20008, USA

[3]Center for Species Survival, Smithsonian National Zoo & Conservation Biology Institute, Front Royal, 22630, USA

[4]School of Integrative Studies, George Mason University, Fairfax, 22030, USA

Corresponding author: Morgan Bragg, mbragg2@gmu.edu, 703-993-1043

Canine IBD Activity Index, Inflammatory Bowel Disease scoring questionnaire
This scoring system, developed by Jergens *et al.*, (2003) for domestic dog, will give us a standardized way to look at the symptomatic presentation of IBD in red wolves. Please score each symptom/sign at the time of the fecal collection with 0 being the least severe and 3 being the most severe.

1. Attitude/activity =_____
    a. 0=normal
    b. 1=slightly decreased
    c. 2= moderately decreased
    d. 3=severely decreased

2. Appetite =______
    a. 0=normal
    b. 1= slightly decreased
    c. 2= moderately decreased
    d. 3= severely decreased

3. Vomiting=______
    a. 0=none
    b. 1=mild (1time/week)
    c. 2= moderate (2-3 times/week)
    d. 3=severe (>3 times/week)

4. Stool consistency=__
    a. 0=normal= slightly soft feces
    b. 1=soft feces or fecal blood mucus or both
    c. 2=very soft feces
    d. 3=watery diarrhea

5. Stool frequency=______
    a. 0=normal
    b. 1=slightly increased (2-3 times/day)
    c. 2= moderately increased (2-5 times/day)
    d. 3= severely increased (>5 times/day)

6. Weight loss=_____
    a. 0=none
    b. 1=mild (<5% loss)
    c. 2= moderate (5-10% loss)
    d. 3=severe (>10% loss)

Supplementary Table 1. Estimate of coefficients of the full linear model investigating the impact of environmental variables on the log10 transformed weekly average FGM concentrations of red wolves (n=14).

	**log10(Weekly_avg)**		
Predictors	*Estimates*	*CI*	*p*
(Intercept)	-0.43	-3.44 – 2.58	0.777
Diet type [Mixed]	-0.69	-0.89 – -0.48	**<0.001**
CIBDAI	-0.03	-0.09 – 0.02	0.232
Sex [M]	0.07	-0.04 – 0.18	0.206
Public access [Self]	-0.29	-0.90 – 0.33	0.356
Public access [Staff]	-0.64	-1.38 – 0.10	0.092
Density [log10]	0.99	0.12 – 1.87	**0.027**
**Random Effects**			
σ^2	0.09		
$\tau_{00\ Animal}$	0.00		
$\tau_{00\ Facility}$	0.05		
ICC	0.37		
N_{Animal}	14		
$N_{Facility}$	7		
Observations	301		
Marginal R^2 / Conditional R^2	0.355 / 0.596		

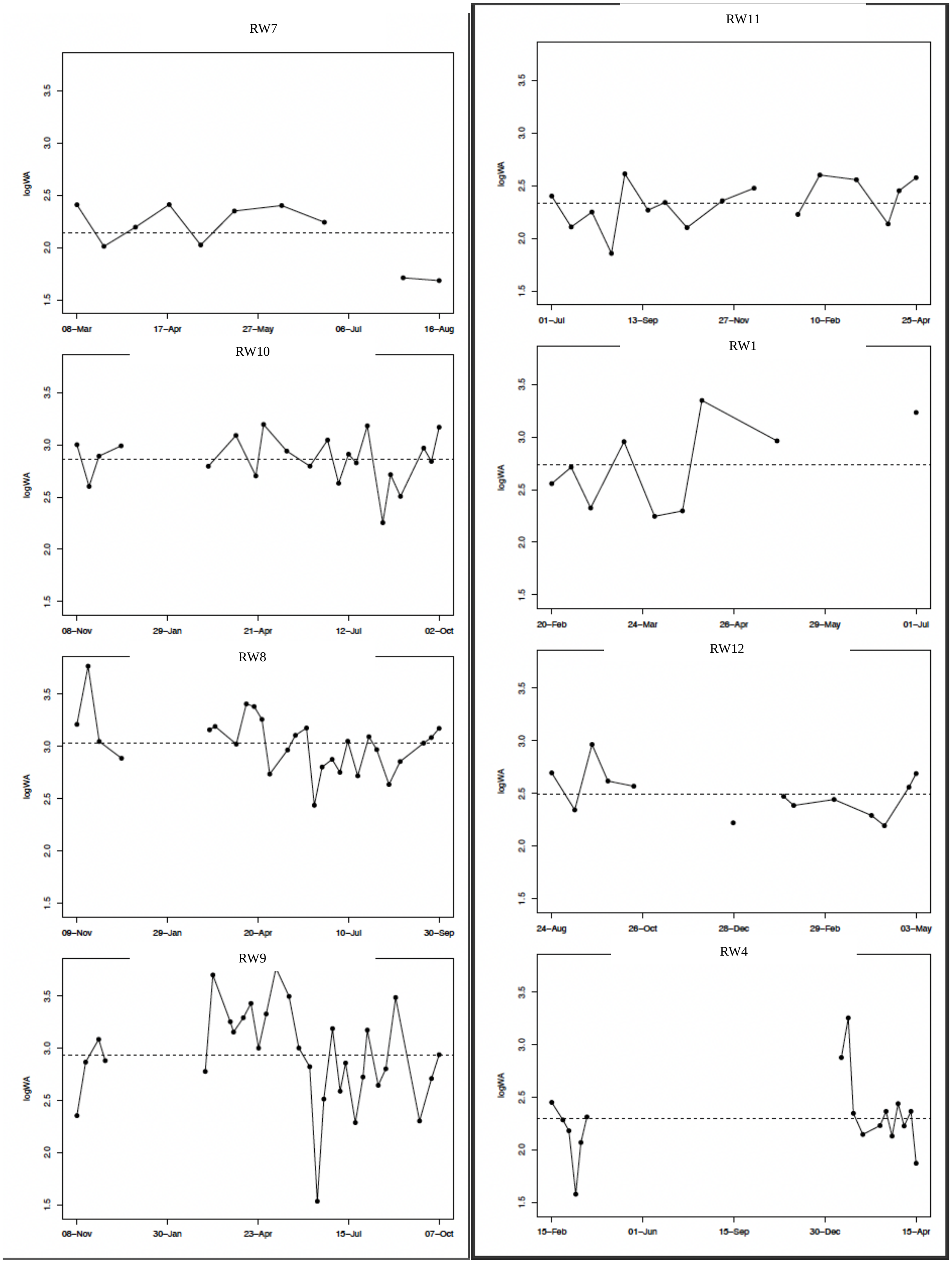
RW7
logWA
1.5 2.0 2.5 3.0 3.5
08–Mar 17–Apr 27–May 06–Jul 16–Aug
RW10
logWA
08–Nov 29–Jan 21–Apr 12–Jul 02–Oct
RW8
logWA
09–Nov 29–Jan 20–Apr 10–Jul 30–Sep
RW9
logWA
08–Nov 30–Jan 23–Apr 15–Jul 07–Oct
RW11
logWA
01–Jul 13–Sep 27–Nov 10–Feb 25–Apr
RW1
logWA
20–Feb 24–Mar 26–Apr 29–May 01–Jul
RW12
logWA
24–Aug 26–Oct 28–Dec 29–Feb 03–May
RW4
logWA
15–Feb 01–Jun 15–Sep 30–Dec 15–Apr

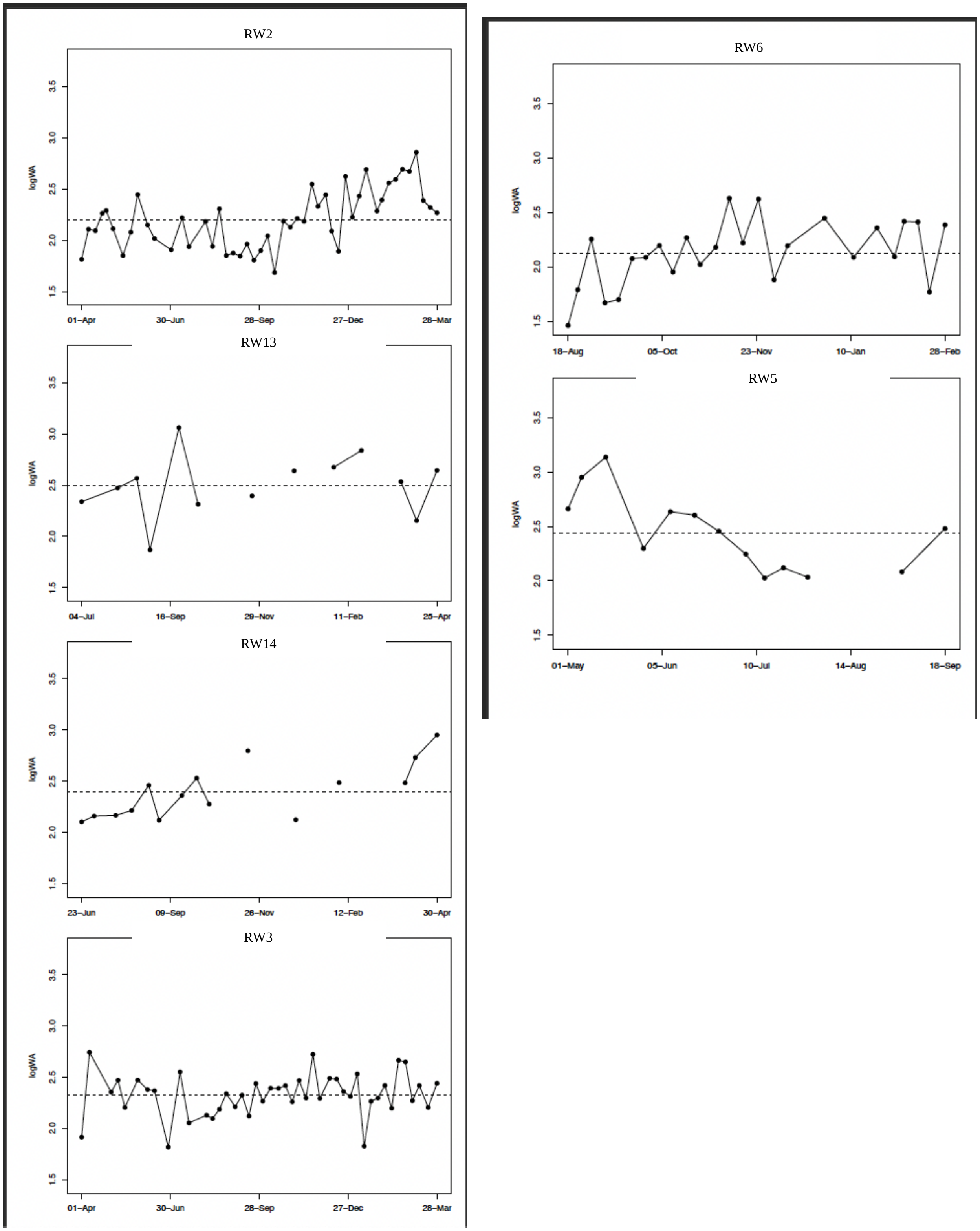

Supplementary Figure 1. Longitudinal profiles of log10 weekly average fecal glucocorticoid metabolite (logWA) concentrations for all fourteen red wolves. The dashed line represents the mean log10 weekly average fecal glucocorticoid metabolite concentration for each individual.
